# Immunomodulatory and Anti-Inflammatory Activities of Chicken Cathelicidin-2 Derived Peptides

**DOI:** 10.1371/journal.pone.0147919

**Published:** 2016-02-05

**Authors:** Albert van Dijk, Mandy van Eldik, Edwin J. A. Veldhuizen, Hanne L. M. Tjeerdsma-van Bokhoven, Marcel R. de Zoete, Floris J. Bikker, Henk P. Haagsman

**Affiliations:** 1 Division of Molecular Host Defence, Department of Infectious Diseases and Immunology, Faculty of Veterinary Medicine, Utrecht University, Utrecht, The Netherlands; 2 Department of Immunobiology, Yale School of Medicine, New Haven, Connecticut, United States of America; 3 Department of Oral Biochemistry, Academic Centre for Dentistry Amsterdam, University of Amsterdam, VU University Amsterdam, Amsterdam, The Netherlands; Nanyang Technological University, SINGAPORE

## Abstract

Host Defence Peptides and derived peptides are promising classes of antimicrobial and immunomodulatory lead compounds. For this purpose we examined whether chicken cathelicidin-2 (CATH-2)-derived peptides modulate the function and inflammatory response of avian immune cells. Using a chicken macrophage cell line (HD11) we found that full-length CATH-2 dose-dependently induced transcription of chemokines CXCLi2/IL-8, MCP-3 and CCLi4/RANTES, but not of pro-inflammatory cytokine IL-1β. In addition, CATH-2 efficiently inhibited IL-1β and nitric oxide production by HD11 cells induced by different sources of lipopolysaccharides (LPS). N-terminal truncated CATH-2 derived peptides maintained the capacity to selectively induce chemokine transcription, but despite their high LPS affinity several analogs lacked LPS-neutralizing capacity. Substitution of phenylalanine residues by tryptophan introduced endotoxin neutralization capacity in inactive truncated CATH-2 derived peptides. In contrast, amino acid substitution of phenylalanine by tyrosine abrogated endotoxin neutralization activity of CATH-2 analogs. These findings support a pivotal role for aromatic residues in peptide-mediated endotoxin neutralization by CATH-2 analogs and were shown to be independent of LPS affinity. The capacity to modulate chemokine production and dampen endotoxin-induced pro-inflammatory responses in chicken immune cells implicates that small CATH-2 based peptides could serve as leads for the design of CATH-2 based immunomodulatory anti-infectives.

## Introduction

Worldwide, antibiotics are extensively used by the livestock industry in veterinary therapy and as a feed additive to promote animal growth, especially in the poultry and swine industry, and it is estimated to exceed 50% of the total antibiotics use in some countries [1]. The antibiotics used in veterinary medicine are structurally related to antibiotics commonly used for human therapy [2]. Micro-organisms have demonstrated to develop resistance against conventional antibiotics [3] and infections with multi-drug resistant bacteria are an increasingly common challenge in hospitals while the number of new antimicrobials in development is low. Hence, to reduce the use of conventional antibiotics in veterinary medicine, alternative strategies are needed that suppress outbreaks of infectious diseases in farm animals.

A promising approach is the use of host defense peptides (HDPs) such as cathelicidins and defensins. HDPs are capable of killing a wide variety of micro-organisms, including multidrug-resistant bacteria, yeast, fungi, protozoa and viruses. In contrast to conventional antibiotics, HDP-mediated killing of micro-organisms occurs via multiple mechanisms attacking both the cell membrane and intracellular targets [4] and despite millions of years of co-evolution, most micro-organisms remain highly susceptible to HDP-mediated killing [5]. Moreover, HDPs exhibit a diversity of immune-related functions. Some HDPs, such as LL-37 inhibit LPS-induced pro-inflammatory cytokine production [6,7] and protect against development of endotoxin shock *in vivo* [8]. HDPs may act directly [9] or indirectly by selectively inducing chemokine production in immune cells [8] and recruitment of other immune cells to the site of infection. At the same time, HDPs may enhance antigen uptake and presentation [10] and inhibit apoptosis of neutrophils and macrophages [11,12]. Thus, in addition to direct antimicrobial activities, HDP-derived peptides may boost the innate and adaptive immune system leading to prevention and improved resolution of infectious diseases [13].

Cathelicidin-2 (CATH-2) is a highly cationic (11+) chicken heterophil-derived peptide with antibacterial and antifungal activities [14]. We previously demonstrated that CATH-2 inhibits LPS-induced production of TNFα, IL-6, IL8 and IL-10 and induces expression of Monocyte Chemotactic Protein 1 (MCP-1) in human peripheral blood mononuclear cells (PBMCs) [13]. The immunomodulatory effects of CATH-2 derived peptides on avian immune cells have not been studied.

CATH-2 has the potential to serve as a paradigm for the development of anti-infectives in poultry with immunomodulatory and/or antibacterial activities. Therefore, we examined the immunomodulatory and anti-inflammatory effects of CATH-2 derived peptides on the avian macrophage-like HD11 cell line. We demonstrate that CATH-2 and several truncated analogs thereof selectively induce chemokine transcription in HD11 cells and inhibit LPS-induced IL-1β and nitric oxide production. We further show that the property to neutralize LPS can be modulated by aromatic amino acid substitution.

## Materials and Methods

### Peptide synthesis

Peptides (Table 1) were synthesized by Pepscan (Lelystad, The Netherlands) and at ACTA (Amsterdam, The Netherlands). The peptides were purified by reverse-phase HPLC and their purity (≥95%) was confirmed by mass spectrometry.

**Table 1:** Amino acid sequences and minimal inhibitory concentrations of CATH-2 analogs.

Peptide	Amino acid sequence	Length /Charge	MIC (μM)
C(1–15)	**RFGRFLRKIRRFRPK**	15, +8	16.7 ± 5.8
CATH-2	**RFGRFLRKIRRFRPKVTITIQGSARF-NH**_**2**_	26, +10	5.0 ± 0.0
C(1–21)	**RFGRFLRKIRRFRPKVTITIQ-NH**_**2**_	21, +9	13.3 ± 5.8
C(4–21)	**RFLRKIRRFRPKVTITIQ-NH**_**2**_	18, +8	6.7 ± 2.9
C(5–21)	**FLRKIRRFRPKVTITIQ-NH**_**2**_	17, +7	8.3 ± 5.8
C(7–21)	**RKIRRFRPKVTITIQ-NH**_**2**_	15, +7	8.3 ± 2.9
C(8–21)	**KIRRFRPKVTITIQ-NH**_**2**_	14, +6	11.7 ± 7.6
C(9–21)	**IRRFRPKVTITIQ-NH**_**2**_	13, +5	8.3 ± 2.9
C(10–21)	**RRFRPKVTITIQ-NH**_**2**_	12, +5	6.7 ± 2.9
C(11–21)	**RFRPKVTITIQ-NH**_**2**_	11, +4	8.3 ± 2.9
C(1–21)W3	**R****W****GR****W****LRKIRR****W****RPKVTITIQ-NH**_**2**_	21, +9	13.3 ± 5.8
C(4–21)W2	**R****W****LRKIRR****W****RPKVTITIQ-NH**_**2**_	18, +8	8.3 ± 2.9
C(7–21)W	**RKIRR****W****RPKVTITIQ-NH**_**2**_	15, +7	13.3 ± 5.8
C(10–21)W	**RR****W****RPKVTITIQ-NH**_**2**_	12, +5	11.7 ± 7.6
C(1–21)Y3	**R****Y****GR****Y****LRKIRR****Y****RPKVTITIQ-NH**_**2**_	21, +9	10.0 ± 0.0
C(4–21)Y2	**R****Y****LRKIRR****Y****RPKVTITIQ-NH**_**2**_	18, +8	10.0 ± 0.0
C(7–21)Y	**RKIRR****Y****RPKVTITIQ-NH**_**2**_	15, +7	11.7 ± 7.6
C(10–21)Y	**RR****Y****RPKVTITIQ-NH**_**2**_	12, +5	16.7 ± 5.8

Length is measured in amino acids and substituted amino acid residues are underlined. Antibacterial activity of CATH-2 analogs against avian pathogenic *E*. *coli* in colony count assays. Means ± SEM for 3 independent experiments.

### Antibacterial activity

Avian pathogenic Escherichia coli O78 (field isolate, Zoetis, Kalamazoo, MI) was cultured in Mueller-Hinton broth at 37°C. Overnight grown cultures were diluted to a density of 2×10^6^ CFU/ml in Mueller-Hinton broth (non cation-adjusted, Oxoid Ltd., Hampshire, UK) and mixed in a 96 wells polypropylene microwell plate at a 1:1 ratio with peptide (final concentration: 1.25 to 40 μM). After 3 h incubation samples were serially diluted in medium, plated on trypton soy agar (Oxoid Ltd., Hampshire, UK) media and counted after 24 h incubation at 37°C.

### Lipopolysaccharide isolation

*Neisseria meningitidis* strain H44/76 was cultured on GC agar plates (Difco, Basingstoke, United Kingdom) supplemented with Vitox (Oxoid Ltd., Hampshire, UK) in an atmosphere of 5% CO_2_ in air at 37°C. *Campylobacter jejuni ATCC11168* was cultured on blood agar base II (Oxoid) plates containing 5% saponized horse blood (Biotrading, Mijdrecht, The Netherlands) at 37°C under microaerophilic conditions (5% O_2_, 10% CO_2_ and 85% N_2_). *Escherichia coli O111*:*B4* was grown on Luria-Bertani agar plates (Biotrading) at 37°C. A wild type *Salmonella enteritidis* 706 strain was grown in Todd-Hewitt broth at 37°C. LPS from was isolated by hot-phenol extraction as described by Westphal and Jann [15]. LPS was quantified using the 3-deoxy-d-manno-2-octulosonic acid assay and 3-deoxy-d-manno-2-octulosonic acid-NH_3_ as standard [16]. Ultrapure *Salmonella minnesota* R595 LPS was obtained from InvivoGen (Toulouse, France).

### LPS binding affinity

Dansyl-polymyxin B was prepared and quantitated as described by Schindler and Teuber [17]. Subsequently, the capacity of peptides to displace dansyl-labeled polymyxin B complexed to *S*. *minnesota* LPS was determined according to Moore *et al*. [18].

### Cell culture

HD11 cells [19] were a kind gift of dr. Jos van Putten (department of Infectious diseases and Immunology, Utrecht University, The Netherlands). HD11 cells were seeded in 96 wells tissue culture treated plates (1×10^5^ cell/mL) and incubated for 18 h at 37°C (5% CO_2_) before treatment. For peptide-induced effects cells were treated with RPMI-1640 medium containing 20 μM peptide during 4 h or 24 h. In LPS neutralization experiments, final concentrations of 50–100 ng/ml LPS were pre-incubated with or without 20 μM peptide for 30 min at 37°C (5% CO_2_), applied to the cells and incubated for 4 h. To examine neutralization of LPS-primed cells HD11 cells were exposed for 30 min to 100–1000 ng/ml *S*. *minnesota* LPS, washed once and incubated during 4 h in the absence or presence of 20 μM Peptide. Supernatants were collected for the determination of nitric oxide production (24 h incubations only). Remaining cells were lysed in lysis buffer and total RNA was isolated and purified using a High Pure RNA isolation kit (Roche, Mannheim, Germany) according the manufacturer's recommendations. RNA quantity and purity were tested using a Nanodrop ND-1000 spectrophometer (Nanodrop Technologies, Wilmington, DE). Cytotoxicity of peptides was determined using the cell viability reagent WST-1 (Roche) as described previously [13], and by measuring LDH release after 24 h exposure. LDH activities in supernatant cell fractions were measured using the Cytoxicity Detection Kit PLUS (Roche) according the manufacturers recommendations and expressed as the % of released LDH relative to the total LDH activity.

### Real time PCR

RNA (250 ng) was reverse transcribed using an iScript cDNA synthesis kit (Bio-rad laboratories (Hemel Hempstead, UK) according to the manufacturers’ instructions. Primers and probes were designed and produced by Eurogentec (Seraing, Belgium) (Table 2). Quantitative real time PCR was performed on a Bio-rad MyiQ system using a qPCR Mastermix (Eurogentec) and 400 nM of each primer and probe. Reactions were performed as follows: 3 min at 95°C; 40 cycles: 10 s at 95°C, 30 s at 60°C and 30 s at 72°C. Relative gene expression levels were normalized against the expression levels of the house keeping genes GAPDH and 28S.

**Table 2:** Primers used for quantitative real time PCR.

Gene	Reference	Primer sequence	Product (bp)
**28S**	X59733	F: 5’-GGCGAAGCCAGAGGAAACT-3’	62
		R: 5’-GACGACCGATTTGCACGTC-3’	
		P: 5’-(FAM)-AGGACCGCTACGGACCTCCACCA-(TAMRA)-3’	
**GAPDH**	NM 204305	F: 5’-GCCGTCCTCTCTGGCAAAG-3’	73
		R: 5’-TGTAAACCATGTAGTTCAGATCGATGA-3’	
		P: 5’-(FAM)AGTGGTGGCCATCAATGATCCC-(BHQ1)-3’	
**IL-1β**	NM 204524	F: 5’-GCTCTACATGTCGTGTGTGATGAG-3’	80
		R: 5’-TGTCGATGTCCCGCATGA-3’	
		P: 5’-(FAM)CCACACTGCAGCTGGAGGAAGCC-(BHQ1)-3’	
**CXCLi2**	NM 205498	F: 5’-GCCCTCCTCCTGGTTTCA-3’	68
		R: 5’-CGCAGCTCATTCCCCATCT-3’	
		P: 5’-(FAM)-TGCTCTGTCGCAAGGTAGGACGCTG-(BHQ1)-3’	
**MCP-3**	Kaiser *et al*.,	F: 5’-CTGCTGCTTCTCCTATGTTCAAC-3’	126
	2005 [20]	R: 5’-ACACATATCTCCCTCCCTTTCTTG-3’	
		P: 5’-(FAM)-ACCTCATTGCCTCCGCCTACATCACCA-(BHQ1)-3’	
**CCLi4**	AY037859	F: 5’-CCCTCTCCATCCTCCTGGTT-3’	66
		R: 5’-TATCAGCCCCAAACGGAGAT-3’	
		P: 5’-(FAM)-CCGCCCTCTTCCCTCAAGCCTC-(BHQ1)-3’	

### Nitric oxide production

Nitric oxide production was assessed as the accumulation of nitrite (NO_2_^−^) in cell supernatants during a 24 h incubation period. Nitrite concentrations were determined using a colorimetric reaction with the Griess reagent using (from 1 to 50 μM) sodium nitrite dissolved in water as standards. Briefly, cell culture supernatants were mixed with an equal volume of 1% sulfanilamide (dissolved in 2.5% phosphoric acid) and incubated for 5 min. The same volume of 0.1% *N*-(1-naphthyl) ethylenediamine dihydrochloride was added and incubated for 5 min. The absorbance was measured at 520 nm using a 96-well microplate reader (FLUOstar Omega, BMG labtech).

### Statistical analysis

Statistical analyses were performed using SPSS version 16.0 statistical software (SPSS inc., Chicago, IL) with one-way analysis of variance (ANOVA) and Dunnett post hoc tests.

## Results

### Antibacterial and cytotoxic activities of CATH-2-derived peptides

**Fig 1:**
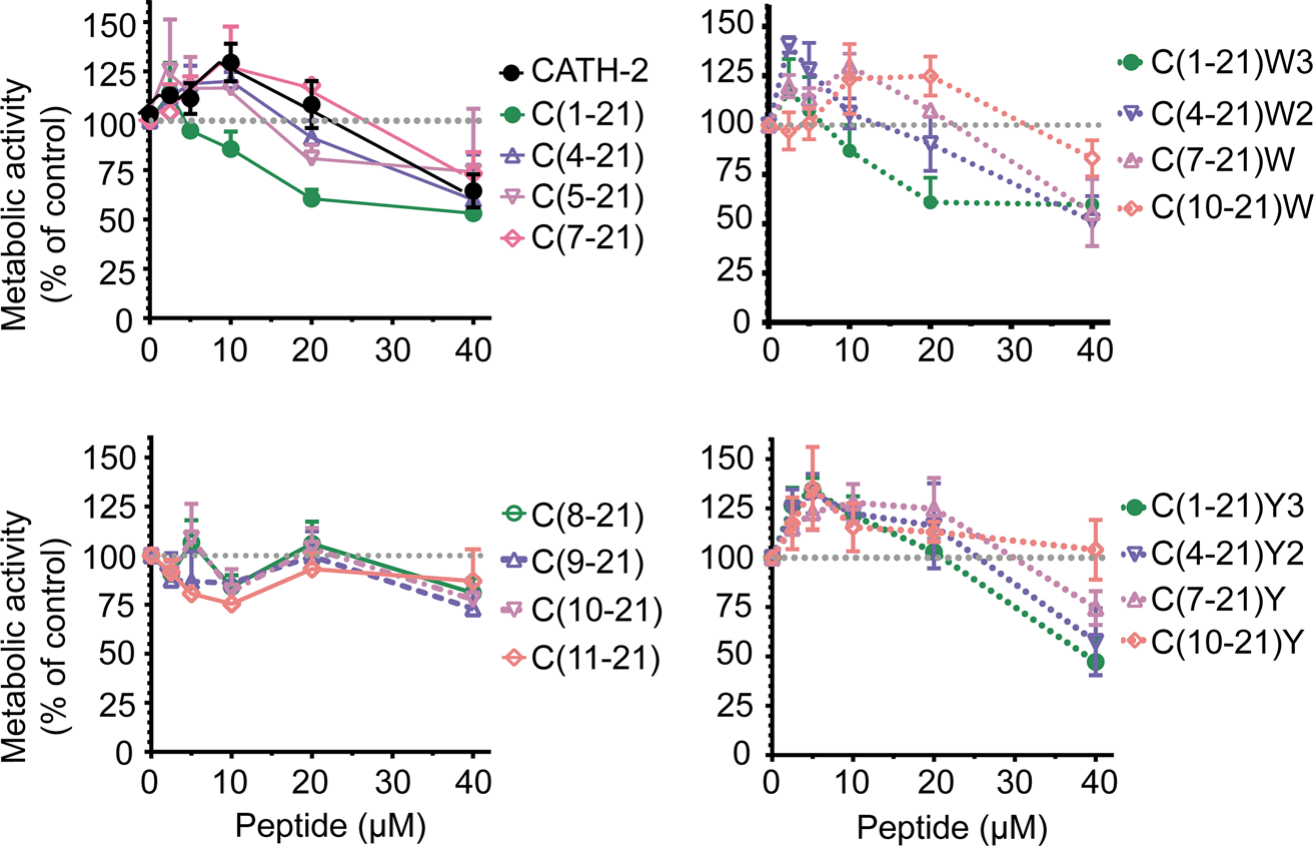
Metabolic activity of CATH-2 derived peptide treated HD11 cells. Metabolic activity of HD11 determined by WST-1 assays after 24 h stimulation with CATH-2 derived peptides (2.5–20 μM). Shown are means ± SEM for at least 3 independent experiments.

**Fig 2:**
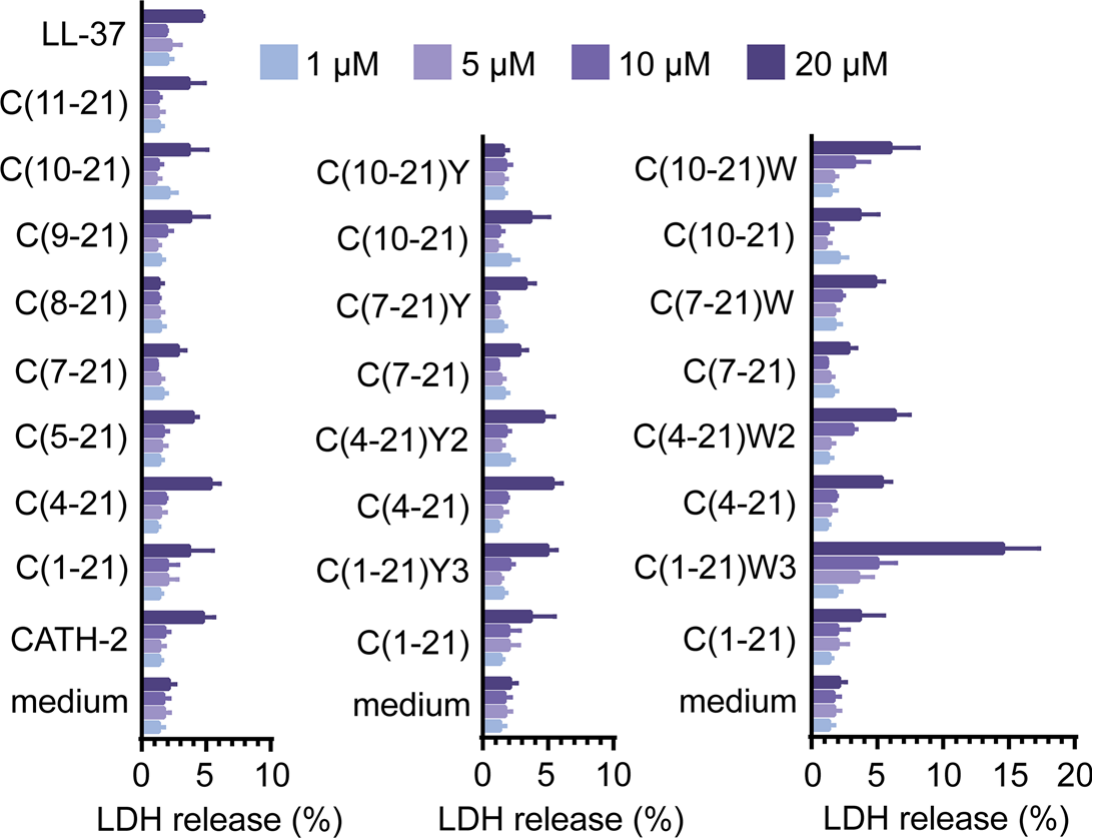
Peptide-induced LDH release from HD11 cells. The percentage of lactate dehydrogenase (LDH) released from HD11 cells during 24 h exposure to medium containing CATH2 derived peptides (1–20 μM). Shown are means ± SEM for at least 3 independent experiments.

To determine the effect of truncation and amino acid substitution on the antibacterial activity minimal inhibitory concentrations (MIC) were determined using colony count assays. All truncated and substituted peptides exhibited antibacterial activity against avian pathogenic *E*. *coli* O78 (Table 1); bacterial survival was reduced to below the detection limit in the presence of 5–20 μM peptide. To determine the toxicity of CATH-2 analogs HD11 cells were exposed for 24 h to different peptide concentrations. Most peptides did not affect HD11 metabolic activity up to peptide concentrations of 20 μM (Fig 1). At 40 μM, HD11 metabolic activities decreased considerably for all peptides except peptide C(10–21)[F12Y]. The peptide-induced LDH release from HD11 cells during 24 h exposure corresponded well with the metabolic activities of HD11 cells (Fig 2); less than 5% LDH release occurred for most peptides at 20 μM.

### Full-length CATH-2 peptide selectively induces chemokine transcription in HD11 cells

**Fig 3:**
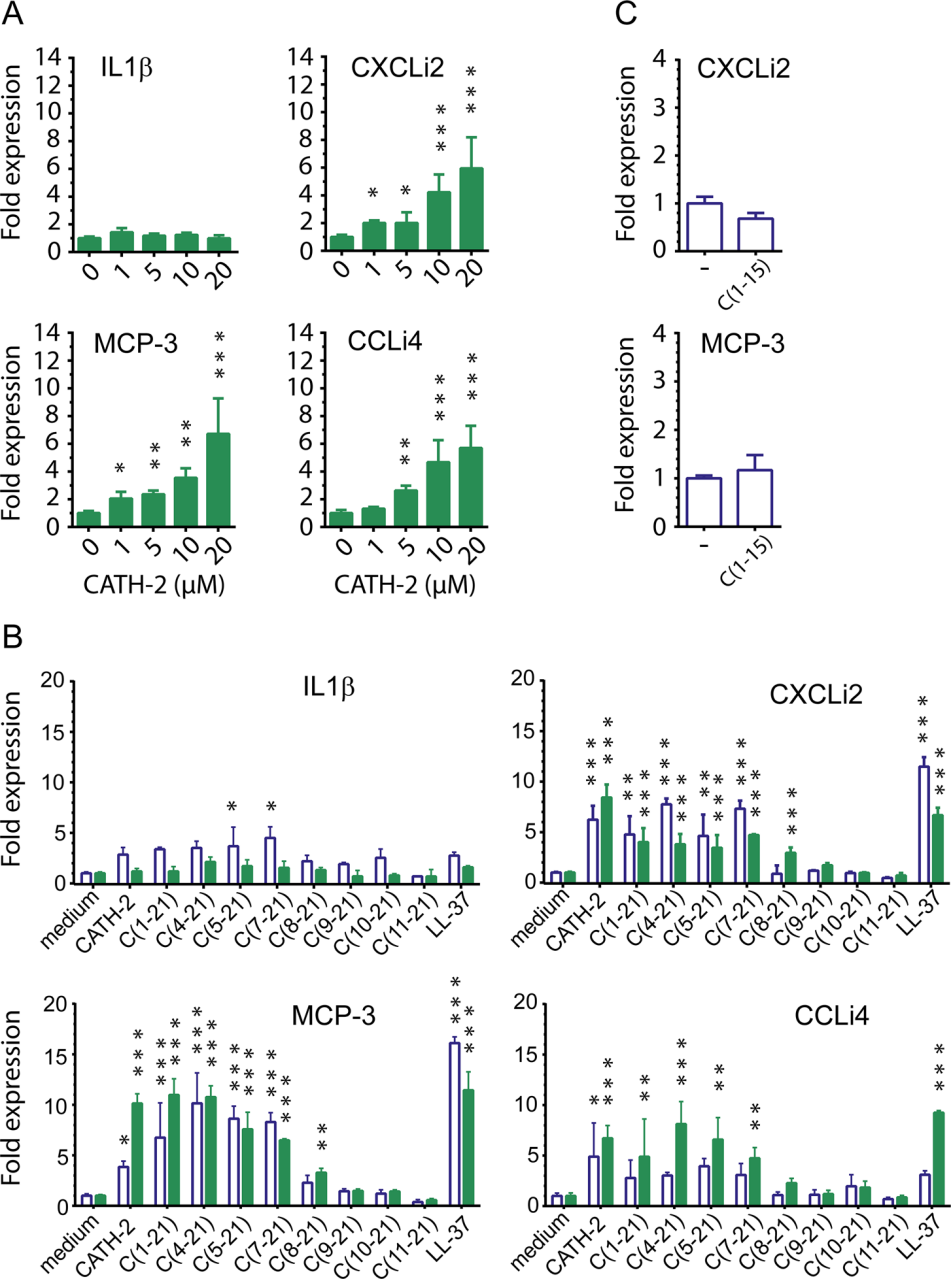
Induction of chemokine transcription in HD11 cells by truncated CATH-2 analogs. CXCLi2, MCP-3, CCLi4 and IL-1β transcription in HD11 cells treated with CATH-2 derived peptides (1 to 20 μM). Transcription was measured by real time QPCR analysis after 4 h (open bars) or 24 h (closed bars) of stimulation. **a** CATH-2 dose-dependently induced transcription of chemokines chCXCLi2/IL-8, MCP-3 and chCCLi4/RANTES, but not of pro-inflammatory cytokine IL-1β. **b** The capacity to induce chemokine transcription was maintained for N-terminally truncated C(1–21) analogs up to 14 amino acid residues during 24 h exposure. **c** No chemokine induction was observed when cells were exposed (4 h) to peptide C(1–15). Data from 3 to 4 independent experiments; means ± SEM * p<0.05, ** p<0.01, *** p<0.001.

To examine if CATH-2 analogs induce chemokine production in chicken macrophages, HD11 cells were exposed to different doses of peptide. CATH-2 was found to dose-dependently increase transcription of chemokines CXCLi2/IL-8, MCP-3 and CCLi4/RANTES in HD11 cells, whereas pro-inflammatory cytokine IL-1β was not induced (Fig 3A). Induced transcription of CXCLi2 and MCP-3 was observed with CATH-2 concentrations as low as 1 μM. To determine the minimal molecular size required for the immunomodulatory activity of CATH-2, truncated analogs were examined for their capacity to induce chemokine transcription in HD11 cells. Brief stimulation (4h) of HD11 cells with 20 μM of N-terminally truncated C(1–21) based peptides induced CXCLi2 and MCP-3 transcription, with a minimal size of 15 amino acid residues (Fig 3B). Prolonged stimulation was needed to induce expression of CCLi4. Further truncation diminished chemokine induction. Peptide C(1–15) comprising the first 15 N-terminal amino acids did not induce chemokine expression (Fig 3C).

### CATH-2 analogs neutralize LPS-induced pro-inflammatory cytokine expression in HD11 cells

**Fig 4:**
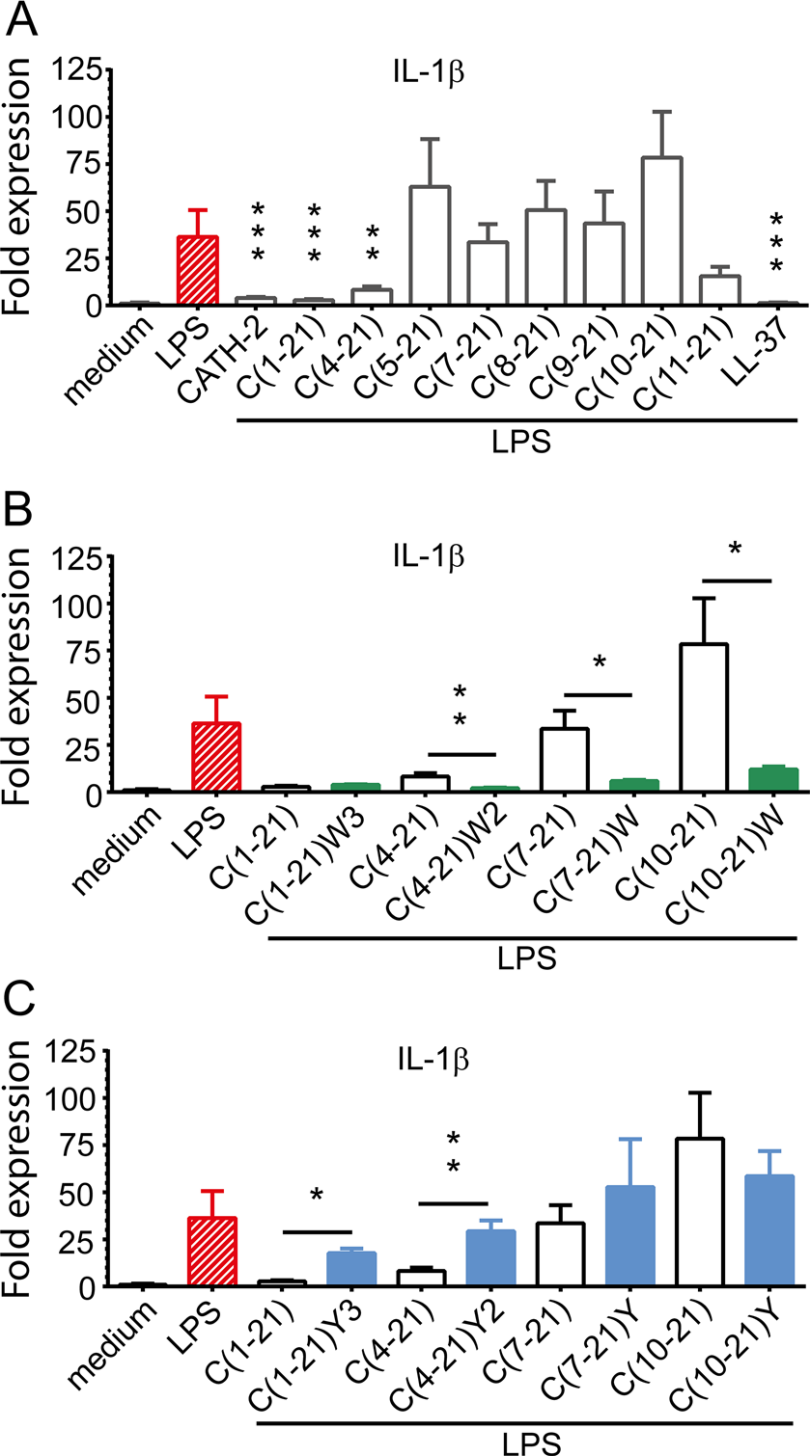
Neutralization of LPS-induced pro-inflammatory cytokine production by CATH-2 analogs. IL-1β transcription in HD11 cells stimulated with *S*. *minnesota* LPS (50 ng/ml) and CATH-2 derived peptides (20 μM). Transcription was measured by real time QPCR analysis after 4 h of stimulation. Amino acid substitutions: W3, [F2W, F5W, F12W]; W2, [F5W, F12W]; W, [F12W]; Y3, [F2Y, F5Y, F12Y]; Y2, [F5Y, F12Y]; Y, [F12Y]. **a** LPS-induced IL-1β transcription in HD11 cells was blocked by full-length peptide and N-terminally truncated C(1–21) analogs up to 18 amino acid residues. **b** Phe/Trp substitution improved the capacity to neutralize LPS-induced IL-1β transcription of peptides C(1–21) and C(4–21) and introduced LPS-neutralizing capacity in formerly inactive peptides C(7–21) and C(10–21). **c** Phe/Tyr substitution of active peptides C(1–21) and C(4–21) abrogated neutralization of LPS-induced IL-1β expression. Data from 3 to 4 independent experiments; means ± SEM. * p<0.05, ** p<0.01, *** p<0.001.

To examine the capacity of CATH-2 analogs to neutralize lipopolysaccharide-induced pro-inflammatory cytokine expression IL-1β mRNA levels were determined after 4 h stimulation of HD11 cells with pre-incubated mixtures of *S*. *minnesota* LPS (50 ng/ml) and CATH-2 analogs (20 μM). LPS-induced IL-1β expression in HD11 cells was blocked by CATH-2 peptide (90.8%) and truncated analogs C(1–21) (93.5%) and C(4–21) (80.5%) (Fig 4A). Further N-terminal truncation completely abrogated the LPS neutralizing potency of peptides. Interestingly, the most truncated peptide, C(11–21), also partially inhibited LPS.

### Aromatic amino acid substitution in CATH-2 analogs modulates neutralization of LPS-induced pro-inflammatory cytokine production

Substitution of Phe by Trp residues in truncated analogs significantly enhanced their capacity to block LPS-induced IL-1β expression in HD11 cells (Fig 4B). Phe/Trp substitution of peptide C(4–21) improved inhibition of LPS-induced IL-1β expression from 80.5% to 95.1%.

Moreover, whereas peptides C(7–21) and C(10–21) lacked LPS-neutralizing activity, their Phe/Trp substituted analogs strongly inhibited LPS-induced pro-inflammatory cytokine production by HD11 cells, *i*.*e*. 86.3% and 71.9%, respectively, compared to the control. In contrast, Phe/Tyr substitution of CATH-2 analogs abrogated their capacity to block LPS-induced IL-1β transcription (Fig 4C). Phe/Trp residues did not consistently alter peptide-induced chemokine expression of HD11 cells (Table 3). Substitution by tyrosine increased overall cytokine transcriptional levels during short exposure, and this effect disappeared when exposure was prolonged.

**Table 3:** Effect of aromatic amino acid substitution on HD11 cytokine expression levels.

	4h stimulation				24 h stimulation			
	IL-1β	CXCLi2	MCP-3	CCLi4	IL-1β	CXCLi2	MCP-3	CCLi4
Medium	1.0±0.1	1.0±0.1	1.0±0.2	1.0±0.3	1.0±0.1	1.0±0.1	1.0±0.1	1.0±0.0
C(1–21)	3.4±0.2	4.8±1.8	6.7±3.4	2.7±1.8	1.9±0.5	4.0±1.4	11.0±1.6	4.9±3.7
C(1–21)W3	3.7±0.3	4.4±1.4	6.2±3.5	1.8±0.2	3.0±0.2	3.5±0.4	15.7±3.4	2.1±0.6
C(1–21)Y3	9.1±6.6	12.6±2.4*	13.7±2.0	6.4±1.2	2.5±0.5	3.3±0.3	8.8±2.1	3.8±0.7
C(4–21)	3.5±0.7	7.8±0.6	10.2±3.0	3.0±0.3	2.1±0.5	3.8±1.0	10.8±1.1	8.1±2.2
C(4–21)W2	5.6±1.4	7.3±1.3	10.1±1.3	3.1±1.0	1.7±0.6	2.5±0.2	7.7±1.1	3.9±0.5
C(4–21)Y2	7.4±4.1	11.6±0.7	12.4±3.0	6.7±1.0	2.1±0.3	4.2±0.5	6.5±0.8	4.6±0.8
C(7–21)	4.5±1.1	7.3±0.8	8.3±0.9	3.1±1.1	1.5±0.7	4.7±0.1	6.5±0.2	4.7±1.0
C(7–21)W	4.1±0.5	4.8±1.0	6.6±2.7	5.5±0.9	0.8±0.1	2.7±0.3	3.4±0.6	1.6±0.5
C(7–21)Y	7.7±4.1	9.4±3.9	11.6±4.5	5.1±1.5	1.8±0.1	4.3±0.3	6.3±0.2	5.4±0.2
C(10–21)	2.5±0.9	0.9±0.1	1.2±0.4	2.0±1.1	0.8±0.2	1.0±0.1	1.4±0.2	1.8±0.6
C(10–21)W	2.6±0.7	2.5±0.3	3.9±1.4	2.1±1.4	0.8±0.2	1.2±0.0	1.5±0.1	1.0±0.2
C(10–21)Y	1.8±0.1	0.9±0.1	1.4±0.5	1.3±2.0	0.7±0.5	1.1±0.1	1.4±0.2	0.7±0.2

Comparison of peptide-induced gene expression levels relative to that of basal expression levels in the absence of peptide. Amino acid substitutions: W3, [F2W, F5W, F12W]; W2, [F5W, F12W]; W, [F12W]; Y3, [F2Y, F5Y, F12Y]; Y2, [F5Y, F12Y]; Y, [F12Y].

Significance level: *, *p*<0.05.

### CATH-2 analogs neutralize LPS-induced nitric oxide production by HD11 cells

Membrane-perturbing agents may induce expression of iNOS in macrophages leading to nitric oxide production. Therefore, we examined whether CATH-2 analogs induced NO production in HD11 cells. Exposure (24 h) of HD11 cells to 20 μM LL-37 or CATH-2 analogs slightly increased (2-fold; data not shown) NO levels, with the exception of peptide C(11–21). This effect was abolished by Phe/Trp substitution, but not by Phe/Trp substitution.

**Fig 5:**
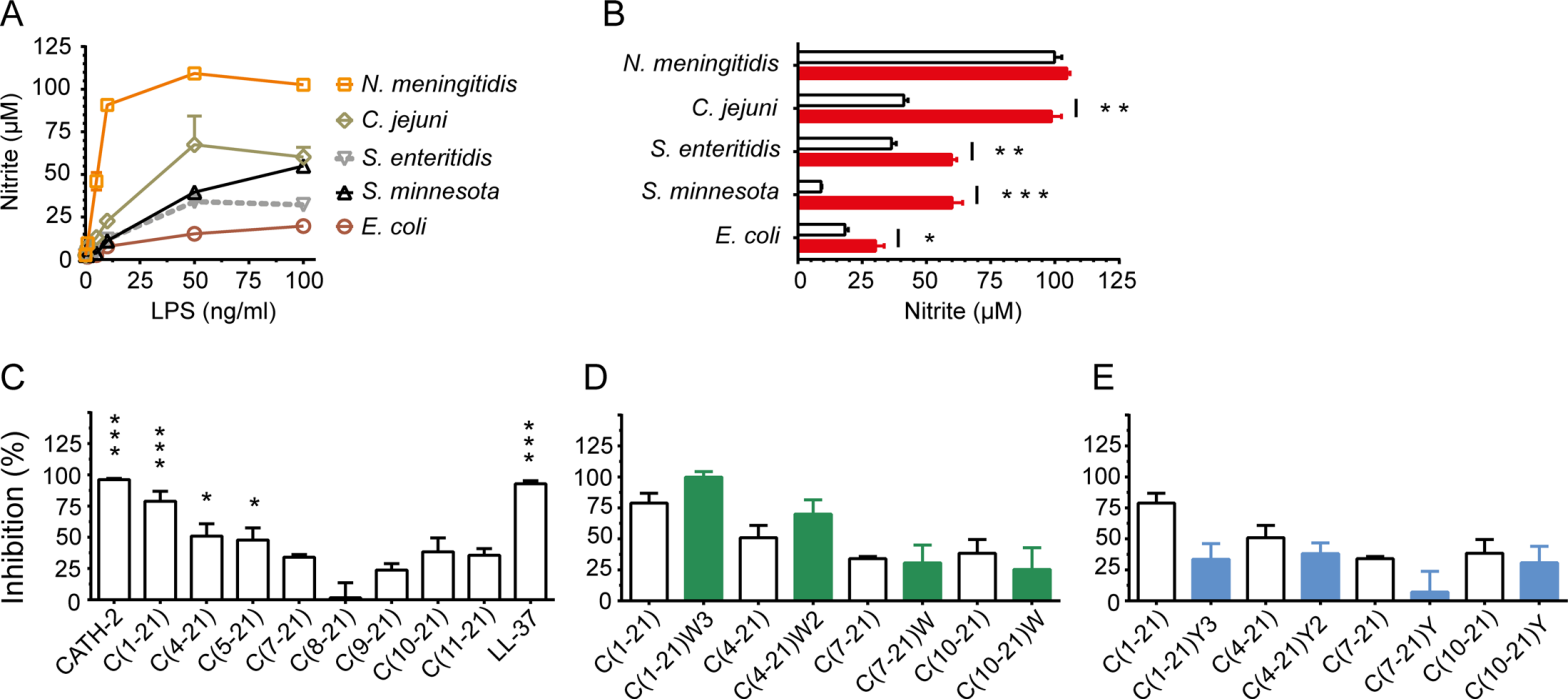
CATH-2 analog neutralization of LPS-induced macrophage nitric oxide production. Nitric oxide production by HD11 cells stimulated with LPS (50 to 100 ng/ml) pre-incubated with CATH-2 derived peptides (20 μM). Nitric oxide (NO) production was measured in supernatants after 24 h incubation using the Griess assay. Amino acid substitutions: W3, [F2W, F5W, F12W]; W2, [F5W, F12W]; W, [F12W]; Y3, [F2Y, F5Y, F12Y]; Y2, [F5Y, F12Y]; Y, [F12Y]. **a** Dose-dependent production of NO by HD11 cells exposed to different sources of LPS. **b** CATH-2 significantly inhibited NO production induced by all tested LPS (100 ng/ml) sources, except wild-type *Neisseria meningitidis* LPS; bars indicate LPS-induced NO production in the absence (closed) and presence (open) of CATH-2 peptide. **c** Inhibition of *S*. *minnesota* LPS-induced (50 ng/ml) NO production by truncated CATH-2 analogs. Production of LPS-induced NO by HD11 cells was significantly reduced in the presence of C(1–21) and N-terminally truncated C(1–21) analogs up to 17 amino acid residues. **d, e** Phe/Trp substitution enhanced inhibition of active peptides C(1–21) and C(4–21), whereas Phe/Tyr substitution abrogated inhibition of LPS-induced NO production by these peptides. Data from 3 to 4 independent experiments; means ± SEM. * p<0.05, ** p<0.01, *** p<0.001.

Nitric oxide (NO) production by macrophages plays a dual role; NO can rapidly react with superoxide to produce peroxynitrate, a reactive oxygen species with potent antimicrobial activity, whereas sustained high levels may lead to tissue damage and cell death [21]. To determine the nitric oxide levels produced by HD11 cells in the presence of LPS, HD11 cells were stimulated for 24 h with different concentrations of LPS derived from different bacterial species. HD11 cells were most susceptible to stimulation by *N*. *meningitidis* H44/76 LPS, *i*.*e*. at 10 ng/ml LPS already induced 83.1% of the maximal NO production (Fig 5A). A moderate response was observed for LPS derived from *C*. *jejuni*, *S*. *minnesota* and *S*. *enteritidis* LPS, *e*.*g*. 50 ng/ml of LPS was needed to produce 61.7%, 36.2% and 31.2%, respectively of the maximal NO production. HD11 production of NO was least responsive to *E*. *coli* O111:B4 LPS.

Next, neutralization of LPS-induced NO production by full-length CATH-2 peptide was examined. For this purpose HD11 cells were stimulated during 24 h with pre-incubated mixtures of LPS (100 ng/ml) and CATH-2 peptide (20 μM). CATH-2 significantly reduced LPS-induced NO production for LPS of all sources except *Neisseria* LPS (Fig 5B). The lack of *Neisseria* LPS inhibition can be explained by the fraction of free LPS. Under the conditions used, CATH-2 most potently inhibited *S*. *minnesota* LPS (85%) of which the residual NO production (10 μM) corresponded to10 ng/ml unbound LPS. A similar unbound fraction of *N*. *meningitidis* LPS, would induce approx. 90 μM NO. Thus, the most likely explanation is that under these conditions the high potency of the unbound fraction of *N*. *meningitidis* LPS was sufficient to obtain a maximal NO production.

Peptide C(1–21) blocked 87.9% of LPS-induced (50 ng/ml) NO production while C(5–21) neutralized NO production by 47.8%. Further truncation diminished the NO neutralization (Fig 5C). Phe/Trp substitution of truncated CATH-2 analogs appeared to increase the inhibition capacity of C(1–21) and C(4–21) (Fig 5D), while Phe/Tyr substitution abrogated inhibition of LPS-induced NO production (Fig 5E).

### Structure of LPS-neutralizing peptides

**Fig 6:**
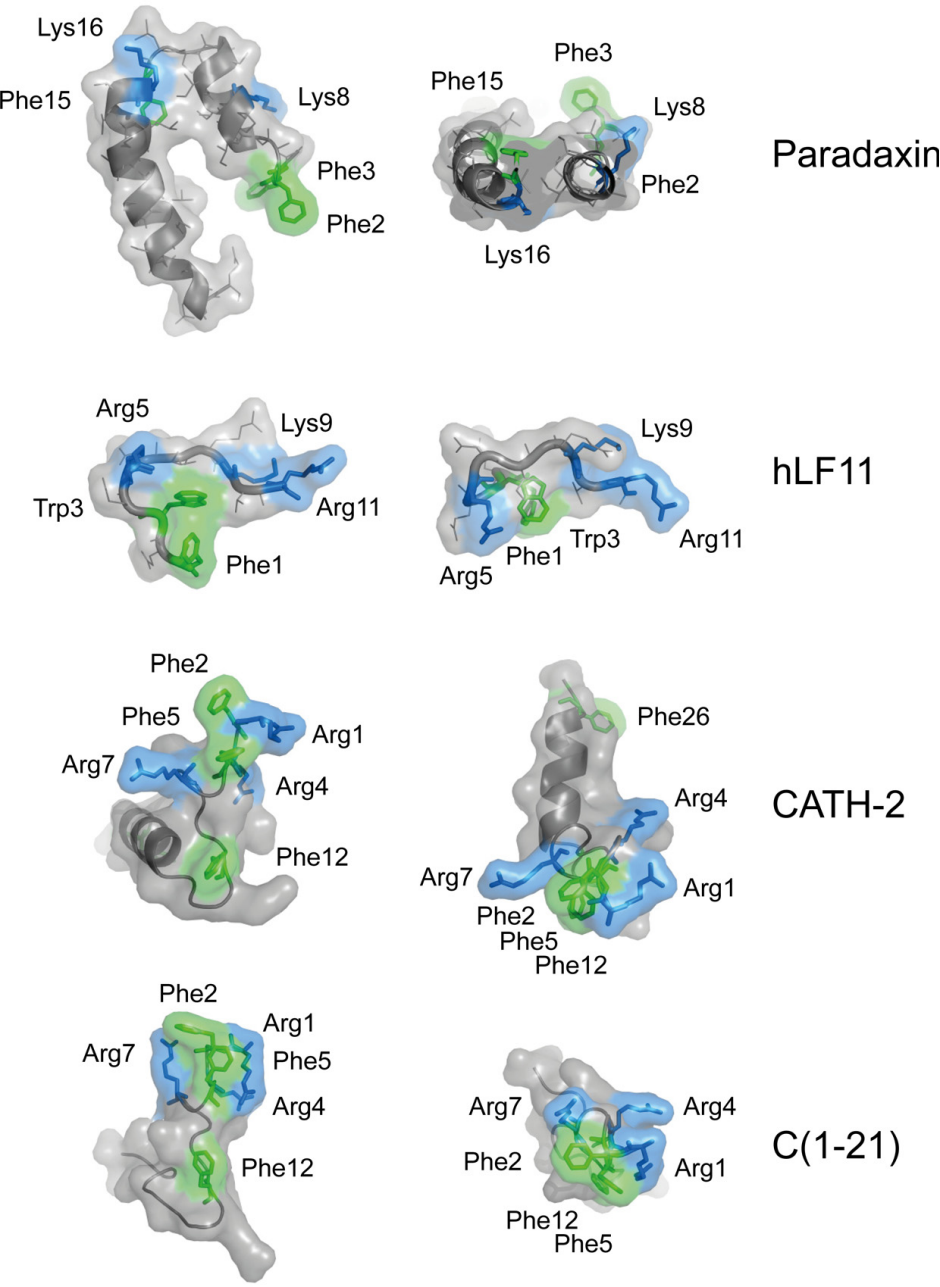
Structural representation of LPS-neutralizing CATH-2 and C1-21 peptide. Representations of the known 3-dimensional structure of paradaxin and human lactoferrin-derived peptide hLF11, cationic peptides with known LPS-binding affinity and the deduced structures of CATH-2 and C(1–21). Side chains reported or deduced to be in closest proximity to lipid A are depicted in green (aromatic side chains) and blue (basic side chains). Representations were adapted from the published structures (RSCB Protein Data bank; http://www.rcsb.org/pdb/home/home.do). The structure of CATH-2 analog C(1–21) was predicted using iTasser.

To visualize the possible mode of CATH-2 analog binding to lipid A, the 3D structures of CATH-2 and C(1–21) were predicted using iTASSER [22] and compared with the configuration of known LPS-binding peptides paradaxin and human lactoferrin (hLF11) when in complex with LPS (Fig 6). Lactoferrin-derived peptide LF11 possesses three basic residues (Arg5, Lys9, Arg11) that match the distance between both glucosamine phosphate groups in lipid A and is known to adopt a T-shaped configuration when binding to LPS [23]. Similarly, the Lys8, Lys16 residues and Phe2, Phe3 residues of paradaxin were shown to be in close proximity of lipid A [24]. The generated models of CATH-2 and C(1–21) indicate that in a hydrophobic environment, e.g. a biological membrane, all Phe residues in these peptides align in the same plane where they may interact with lipid A acyl chains and suggest that N-terminal basic residues Arg^1^, and Arg^4^ Arg^7^ are the most likely residues to interact with lipid head phosphate groups.

### LPS neutralization by CATH-2 analogs does not correlate with LPS binding affinity

Dansyl-polymyxin B (DPmB) experiments were performed to get insight in the relationship between the LPS binding affinity of peptides and their capacity to neutralize LPS. Compared to unlabeled polymyxin B (*I*_*50*_ = 4.19 μM), full-length CATH-2, C(1–21) and C(4–21) displaced DPmB from *S*. *minnesota* LPS at a 20-fold lower concentration (Table 4). However, similar high binding affinities were found for C(7–21) (*I*_*50*_ = 0.33 μM) and C(1–15) (*I*_*50*_ = 0.12 μM) that lacked LPS-neutralization capacity. Further peptide truncation gradually decreased LPS binding capacity and was not affected by aromatic amino acid substitution.

**Table 4:** LPS binding affinity of CATH-2 analogs.

Peptide	I50 (μM)		
	Phe	Phe/Trp	Phe/Tyr
Polymyxin B	3.46 ± 0.17	n.d.	n.d.
LL37	0.28 ± 0.04	n.d.	n.d.
CATH-2	0.18 ± 0.01	n.d.	n.d.
C(1–21)	0.15 ± 0.02	0.16 ± 0.02	0.18 ± 0.01
C(4–21)	0.26 ± 0.05	0.20 ± 0.02	0.22 ± 0.03
C(5–21)	0.67 ± 0.16	n.d.	n.d.
C(7–21)	0.28 ± 0.02	0.25 ± 0.01	0.35 ± 0.06
C(8–21)	1.50 ± 0.09	n.d.	n.d.
C(9–21)	4.89 ± 0.80	n.d.	n.d.
C(10–21)	5.01 ± 1.27	3.09 ± 0.18	5.21 ± 0.99
C(11–21)	42.49 ± 1.83	n.d.	n.d.
C(1–15)	0.13 ± 0.01	n.d.	n.d.

Binding affinity is expressed as the concentration of peptide (μM) needed to displace 50% of Dansyl-labeled polymyxin from *S*. *minnesota* LPS micelles.

**Fig 7:**
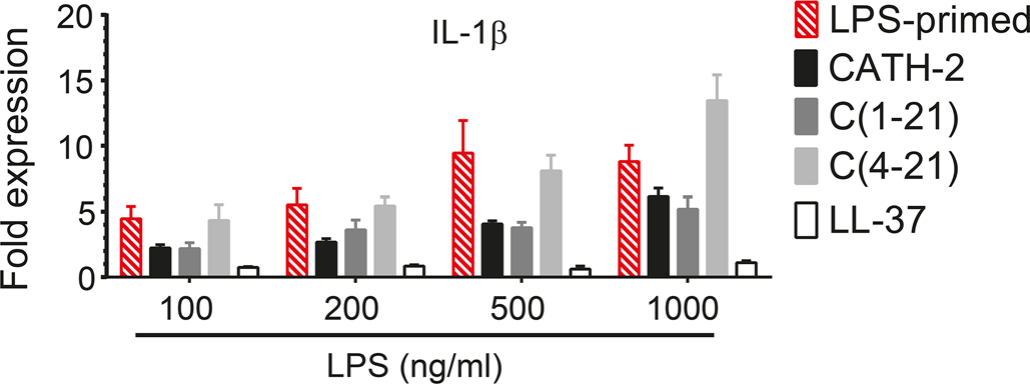
CATH-2 analogs partially neutralize IL-1β transcription in LPS-primed cells. HD11 cells were primed for 30 min with *S*. *minnesota* LPS (100–1000 ng/ml), washed once and incubated during 4h in the presence or absence of 20 μM peptide. IL-1β transcription was measured by real time QPCR analysis. Data from 3 to 4 independent experiments (means ± SEM).

To determine if LPS-neutralization occurred via other mechanisms than direct binding, HD11 cells were primed during 30 minutes with different doses of LPS, washed once to remove exogenous LPS and incubated for 4 h in the absence or presence of peptide. IL-1β transcription induced by LPS priming proved to be partially inhibited by CATH-2 and C(1–21) (p<0.01) and strongly inhibited by LL-37 (p<0.001), whereas no inhibition was observed with C(4–21) (Fig 7).

## Discussion

Cathelicidins [25,26] and cathelicidin-derived peptides, such as IDR-1 [27] and IDR1002 [28], have demonstrated *in vivo* protection against infections by Gram-positive and Gram-negative bacteria. In the case of cathelicidin-derived peptides that lacked direct antimicrobial activity this was found to be associated with the selective induction of chemokines, subsequent recruitment of monocytes and neutrophils, and to be monocyte/macrophage dependent [27]. In this work the immunomodulatory and anti-inflammatory activities of chicken CATH-2 analogs were examined using HD11 cells, a chicken macrophage-like cell line. Full-length CATH-2 peptide and truncated analogs were found to selectively induce CXCLi2/IL-8 and MCP-3 transcription in HD11 cells. These findings corroborate with observations by others for LL-37; induction of MCP-1 expression in murine RAW264.7 cells [8] and induction of MCP-1, MCP-3 and IL-8 via the ERK1/2 and p38 pathways, but not of pro-inflammatory cytokines IL-6, TNF-α or IL-1β, in human monocytes [29]. HD11 cells responded similarly to LL-37 by primarily inducing the transcription of CXCLi2 and MCP-3, suggesting that they may share common signaling pathways.

Experiments with C-terminal and N-terminal truncated analogs of CATH-2 indicated peptide C(7–21) to be the crucial part for peptide-induced chemokine production in HD11 cells. This proved not to be the case for the anti-inflammatory activity of CATH-2 derived peptides. Whereas CATH-2 and truncated analogs C(1–21) and C(4–21) exhibited anti-inflammatory activity, efficiently blocking the LPS-induced IL-1β transcription and NO production by HD11 cells, further truncation abrogated LPS neutralization.

Next we endeavored to enhance the anti-inflammatory properties of smaller CATH-2 analogs while maintaining other immunomodulatory properties. The prominent presence of aromatic moieties in small immunomodulatory peptides [30] and their frequently observed proximity to lipid A acyl chains in peptide-LPS complexes [23,27,31] led us to believe that the position and type of aromatic amino acid may be key factors in enhancing or altering immunomodulatory properties. Indeed, aromatic amino acid residue substitution was found to greatly affect anti-inflammatory activities of truncated CATH-2 analogs, without substantially changing their potency to induce chemokine transcription in HD11 cells. LPS neutralizing capacity could be enhanced in peptide C(4–21) and introduced in inactive peptides C(7–21) and C(10–21) by substitution of the single Phe residue by Trp. Surprisingly, peptide C(1–15) failed to induce chemokine transcription. In addition, C(1–15), previously shown to moderately [13] or weakly [32] inhibit LPS-induced cytokine expression, was under the conditions used not able to block LPS-induced cytokine transcription in HD11 cells (data not shown). Thus, in non-substituted CATH-2 analogs the C-terminal segment (VTITIQ) is pivotal for both immunomodulation and LPS neutralization. However, Phe/Trp substitution of C(1–15) has been shown to introduce significant LPS neutralizing capacity [32]. This can be explained by the behavior of tryptophan in lipid bilayers. Tryptophan side chains and indol groups prefer to localize at the interface of water-associated lipid head groups and acyl chains, probably stabilized by dipole interactions [33,34], suggesting that Phe/Trp-substituted CATH-2 analogs are prone to adopt a different orientation when binding to lipid A. Among aromatic residues Trp is the most polarizable residue and able to form a hydrogen bond via its indol N-H moiety with acyl carbonyl as well as phosphate oxygen groups [35]. Hence, most likely the Phe/Trp substitution in peptide C(10–21) improved LPS neutralization capacity by stabilization of the peptides hydrophobic core to the lipid acyl region. In contrast, substitution of Phe residues by Tyr residues was found to abrogate the LPS neutralizing capacity of ‘active’ CATH-2 analogs. This is comparable to the reported effects of Phe/Trp and Phe/Tyr substitution of Polymyxin B nonapeptide; *i*.*e*. LPS-binding and–neutralization were maintained when Phe was substituted by Trp, but strongly abrogated upon substitution with Tyr [36].

It has been shown that cationic peptides in complex with LPS adopt a configuration in which basic residues interact with lipid A glucosaminoglycan phosphate groups while hydrophobic residues interact with its acyl chains. In the case of the polymyxin B-LPS complex, two positively charged α,γ-diaminobutyric acid (DAB) residues and one Phe residue are necessary for LPS-binding [37]. Structure overlay of the LPS-LF11 complex and *E*. *coli* iron uptake receptor FhuA-LPS complex revealed a conserved structural LPS-binding motif consisting of three basic residues (Arg or Lys) and a single Phe residue [38]. We reasoned that CATH-2 analogs, possessing multiple basic and hydrophobic residues may adopt a similar configuration. Moreover, arginine residues are flanking each aromatic residue, which may aid to stabilize the conformation through formation of intramolecular cation-π interactions between aromatic and arginine residues [39]. Based on the homology found between the deduced C(1–21) peptide configuration and known lipid A binding peptides we consider it likely that this CATH-2 analog interacts with lipid A via its Arg1, Arg4 and Arg7 residues and multiple Phe residues.

However, our results also indicate that LPS neutralization by CATH-2 analogs cannot be explained by LPS binding affinity alone. CATH-2, C(1–21) and LL-37 could partially or completely neutralize IL-1β transcription in LPS-primed HD11 cells, indicating that LPS neutralization by LL-37 and some CATH-2 analogs is in part independent of binding to LPS. Despite its capacity to neutralize LPS when co-incubated, peptide C(4–21) was not able to neutralize LPS-priming induced IL-1β transcription, suggesting a direct binding mode of action for this peptide. Although LPS-binding has been found to be correlated to inhibition of LPS-induced pro-inflammatory cytokine production for several structurally different cationic peptides [6], LPS signaling may be inhibited via alternative mechanisms. For instance, LL-37 and LL-37-derived peptides were shown to partially inhibit LPS signaling through binding to cell surface CD14 [40]. Furthermore, LPS-induced gene expression profiles were found to be differentially altered in the presence of LL-37 and BMAP-27: strongly inhibiting one set of pro-inflammatory genes while the expression levels of other genes, including negative regulators of NF-κB and certain chemokines, were not substantially affected [41]. For LL-37, BMAP-27 and polymyxin B it has also been described that they can inhibit the nuclear translocation of NF-κB subunits p50 and p65, which is pivotal to LPS-induced pro-inflammatory cytokine production [41]. Additionally, LL-37 is known to selectively and directly affect chemokine production via activation of MAPK pathways [29] and it has been suggested that MAP kinase ERK1/2-mediated CREB phosphorylation could inhibit NF-κB activation by competing with CBP/p300 binding to NF-κB subunit p65 [42].

Overall, the immunomodulatory and anti-inflammatory properties of CATH-2 derived peptides demonstrated towards avian macrophages suggest that treatment of poultry with CATH-2 derived peptides may lead to a selective recruitment and activation of avian immune cells while dampening excessive immune responses. We believe that CATH-2 derived peptides, due to their unique properties, could serve as leads for the design of veterinary animal-specific therapeutics.
